# Low-activity [^18^F]-somatostatin receptor (SSTR) imaging using [^18^F]SiTATE on a long axial field-of-view PET/CT scanner

**DOI:** 10.1186/s40658-025-00720-z

**Published:** 2025-02-05

**Authors:** Nils F. Trautwein, Eduardo Calderón, Pia M. Linder, Gerald Reischl, Philippe Driessen, Wenhong Lan, Andreas S. Brendlin, Thorben Groß, Helmut Dittmann, Martina Hinterleitner, Christian la Fougère, Fabian P. Schmidt, Lena S. Kiefer

**Affiliations:** 1 Department of Nuclear Medicine and Clinical Molecular Imaging, University Hospital Tuebingen, Otfried-Mueller-Str. 14, 72076 Tuebingen, Germany; 2 ENETS Center of Excellence, University Hospital Tuebingen, Otfried-Mueller-Str. 14, 72076 Tuebingen, Germany; 3 Werner Siemens Imaging Center, Department of Preclinical Imaging and Radiopharmacy, Eberhard Karls University Tuebingen, Roentgenweg 13, 72076 Tuebingen, Germany; 4 Department of Diagnostic and Interventional Radiology, University Hospital Tuebingen, Hoppe-Seyler-Straße 3, 72076 Tuebingen, Germany; 5 Department of Medical Oncology and Pneumology (Internal Medicine VIII), University Hospital Tuebingen, Otfried-Mueller-Str. 14, 72076 Tuebingen, Germany; 6 DFG Cluster of Excellence 2180 ‘Image-Guided and Functional Instructed Tumor Therapy’ (iFIT), University of Tuebingen, Roentgenweg 11, 72076 Tuebingen, Germany; 7 German Cancer Consortium (DKTK), German Cancer Research Center (DKFZ) Partner Site Tuebingen, Auf der Morgenstelle 15, 72076 Tuebingen, Germany

**Keywords:** LAFOV PET/CT, Total-Body-PET, [^18^F]SiTATE, Low-activity [^18^F]SiTATE PET, [^18^F]-SSTR imaging

## Abstract

**Purpose:**

^18^F-labelled somatostatin receptor tracers have recently gained popularity due to their better spatial resolution, longer half-life and lower costs compared to ^68^Ga-labeled tracers. The aim of this study was to evaluate the impact and limitations of reduced administered activities of [^18^F]SiTATE on image quality, lesion detectability and quantitative PET parameters in a long axial field-of-view (LAFOV) PET/CT scanner.

**Methods:**

Twenty-four patients with histologically confirmed neuroendocrine tumor, who underwent clinically indicated [^18^F]SiTATE PET/CT examination (3.0 MBq/kg, 5 min PET scan time) on a Siemens Biograph Vision Quadra LAFOV PET/CT, were included retrospectively in this study. PET list-mode data were rebinned for shorter frame durations to simulate 5 min scans with lower activities of injected radiotracer. A comparison of image reconstruction in high sensitivity (HS) and ultra-high sensitivity mode (UHS) mode was performed. Subjective image quality, noise and lesion detectability of *n* = 122 lesions were rated using a 5-point Likert scale. The molecular tumor volume (MTV), signal-to-noise ratio (SNR), tumor-to-liver activity concentration ratio (TLR) and standardized uptake values (SUV) were analyzed.

**Results:**

Subjective image quality decreased with simulated reduction of injected activity with generally superior ratings in the UHS mode compared to the HS mode. Despite a reduction to 1 MBq/kg of [^18^F]SiTATE all lesions were still detected while at 0.25 MBq/kg lesion detectability decreased to 70% (HS) and 93% (UHS). Only minor changes in SUV_mean_ and TLR were detected with reduced activity. However, reduced activities led to an increase in SUV_SD_, which in turn caused a decrease in SNR (at 1 MBq/kg: 7.3 in HS and 9.0 in UHS mode and an increase in deviation of the MTV.

**Conclusion:**

Reducing the administered activity of injected [^18^F]SiTATE by 66% to 1 MBq/kg (HS & UHS) is feasible in a LAFOV PET/CT scanner, maintaining clinically diagnostic image quality without statistically significant deviations in PET uptake parameters and MTV. Furthermore, in low activity [^18^F]SiTATE PET/CT, the UHS mode improves image quality and noise as well as lesion detectability compared to HS mode, further reinforcing the clinical benefits of this recently introduced reconstruction mode.

**Supplementary Information:**

The online version contains supplementary material available at 10.1186/s40658-025-00720-z.

## Introduction

Neuroendocrine neoplasms (NENs) are a rare group of tumors that arise from the diffuse neuroendocrine system. NENs are subdivided into well-differentiated neuroendocrine tumors (NETs) and poorly differentiated neuroendocrine carcinomas (NECs) [1]. Well-differentiated NETs are usually characterized by overexpression of somatostatin receptors (SSTR) on their cell surface, representing an excellent target for somatostatin analogues (SSAs) [2]. A major advancement was the development of radiolabeled SSAs, allowing for SSTR imaging by positron emission tomography (PET). SSTR PET imaging has a significant clinical impact on patient management regarding tumor staging, preoperative imaging, treatment selection and surveillance as well as detection of recurrent disease [3]. Within this context, the SSTR-based molecular tumor volume (MTV) may help to predict therapy outcomes and determining treatment courses for patients with NETs [4–6].

^68^Ga radiolabeled DOTA-conjugated SSAs (DOTA-SSA), such as [^68^Ga]-DOTATATE, [^68^Ga]-DOTATOC, and [^68^Ga]-DOTANOC are being considered as the current gold standard for SSTR imaging with PET [7]. However, major disadvantages of ^68^Ga-labelled radiotracers are the high costs of the generators, the shorter half-life of ^68^Ga and the limited activity per elution, which considerably limit the clinical applicability of ^68^Ga-radiotracers in routine practice. In addition, the higher mean positron energy of ^68^Ga compared to ^18^F results in a longer mean positron range (3.5 mm for ^68^Ga and 0.6 mm ^18^F in water), e.g [8]. This leads to a deterioration in spatial resolution and thus image quality and quantification, which is particularly important for the assessment of small lesions. For example, for the Biograph Vision Quadra LAFOV PET/CT scanner with a radial NEMA-NU 2018 spatial resolution of 3.3 mm [9], contrast recovery for a 7.86 mm sphere was reported to decrease from 54% (^18^F) to 37% (^68^Ga) [10]. In contrast, ^18^F is a cyclotron product and has a significantly higher activity yield in combination with a longer half-life. This allows a higher number of SSTR PET examinations per tracer synthesis to be performed in clinical routine [11]. Therefore, it is not surprising that new ^18^F-labeled SSTR tracers, such as [^18^F]AlF-NOTA-octreotide and [^18^F]SiTATE, have recently generated great interest [12, 13].

The major drawback to the wider use of [^18^F]SiTATE to date is the radiosynthesis, which is still in the process of being optimized. The available automated processes so far often suffered from high variations in radiochemical yields, making a reliable scheduling patient care difficult and preventing a broader application in routine clinical practice. Only very recently, radiochemical optimizations showed higher and more stable yields, which should result in an improved tracer supply in the near future. The fluctuating tracer yield of [^18^F]SiTATE and thus potentially low activities can be compensated for, by either longer scan durations or the use of PET scanners with a higher sensitivity. Therefore, recently introduced PET/CT scanners with a long axial field of view (LAFOV) and high sensitivity may enable a more widespread clinical application of these promising ^18^F-labelled SSTR tracers.

In this context, recent studies have demonstrated that in LAFOV PET scanners, a significant reduction of the amount of the injected radiotracer and/or a reduction of PET scan time is possible, while preserving clinically diagnostic image quality [14, 15]. The increase in sensitivity is remarkable, e.g. from 16.4 cps/kBq [16] for the 26.1-cm long Biograph Vision standard axial field of view (SAFOV) PET/CT scanner (Siemens Healthineers, Knoxville, USA) to 83 cps/kBq [9] for the 106-cm long Biograph Vision Quadra LAFOV PET/CT scanner (Siemens Healthineers, Knoxville, USA). Also, the signal-to-noise ratio (SNR) is improved compared to short axial field of view (SAFOV) scanners [17, 18]. The full potential of the Biograph Vision Quadra in terms of sensitivity can be exploited by using its recently introduced ultra-high sensitivity (UHS) mode. The increase of the acceptance angle from 18° (for the standard high sensitivity (HS) mode) to 52° led to a higher sensitivity of 176 cps/kBq [9]. Several studies have recently been performed to assess the impact of the UHS mode on quantification, spatial resolution, partial volume effect and image quality for different isotopes [10, 19]. However, to the best of our knowledge no study on ^18^F-labelled SSTR has been conducted in the Biograph Vision Quadra or any other LAFOV PET scanner.

Therefore, the aim of this study was to systematically assess the impact and limitations of reduced [^18^F]SiTATE injected activities on qualitative and quantitative PET image parameters in patients with NETs on the Biograph Vision Quadra comparing both sensitivity modes. Our hypothesis is that a significant reduction in administered activity is feasible in routine clinical imaging while still providing diagnostic image quality and lesion detectability and maintaining acceptable image noise levels without adversely impacting lesion uptake quantification.

## Materials and methods

### Study cohort

In this retrospective analysis, 24 patients with histology confirmed NET who underwent a clinically indicated [^18^F]SiTATE PET/CT on a LAFOV PET/CT scanner (Biograph Vision Quadra, Siemens Healthineers, Knoxville, TN, USA) were evaluated. The study was based on a prospective PET/CT registry and was approved by the Institutional Review Board of the University Hospital of Tuebingen (#167/2020BO2). Written informed consent was obtained from all patients prior to the PET/CT examination according to the regulations of the German Pharmaceuticals Act § 13.2b. Regarding previous treatment, 21 patients underwent surgery of the primary tumor and 17 patients had been treated with SSA previously. Patient characteristics and details of previous treatments are summarized in Table 1 and per patient in Supplementary Table 1.

**Table 1:** Patient characteristics of the study cohort

Patients characteristics	
Age (y)	66.71 ± 12.43
Sex– n (%)	
Male	11 (46)
Female	13 (54)
Primary tumor site– n (%)	
Midgut	14 (58)
Pancreas	7 (29)
Hindgut	3 (13)
Grading– n (%)	
G1	8 (29)
G2	14 (58)
G3	1 (4)
Gx	1
Previous therapy– n (%)	
Surgery	21 (87.5)
Somatostatin analogues	17 (71)
Chemotherapy	2 (8)
PRRT	11 (46)
SIRT	1 (4)

G = grading, PRRT = peptide receptor radionuclide therapy, SIRT = selective internal radiation therapy

### Tracer synthesis and PET/CT acquisition

[^18^F]SiTATE was prepared in a GMP environment on a TRACERlab MX from GE, Uppsala, Sweden and using cassettes and kits from ABX, Radeberg, Germany [12]. A standard administered activity of 3.0 MBq/kg [^18^F]SiTATE was injected intravenously, and PET image acquisition was started 90 min. p.i [20]. Due to the limited availability of the radiotracer, four patients had to be examined with a reduced administered activity of 0.8 ± 0.1 MBq/kg (Supplementary Table 2). Whole-body scans were obtained in supine position covering an axial extent of 106 cm from the head to the mid-thighs. PET emission data were obtained with a standard acquisition time of 5 min for a single bed position.

### PET image reconstruction

PET reconstruction was performed according to our standard reconstruction protocol, applying an Ordinary-Poisson Ordered-Subsets Expectation-Maximization algorithm with four iterations and five subsets, using point-spread-function modeling and time-of-flight information. The standard correction methods employed by the image reconstruction of the vendor were applied, such as a scatter, random and decay correction. Images were reconstructed with a matrix of 440 × 440 × 645 and a 1.65 × 1.65 × 1.65 mm^3^ isotropic voxel size. No filter was applied. The diagnostic CT scans, which were acquired just before the emission measurements, were used for attenuation correction.

**Fig. 1:**
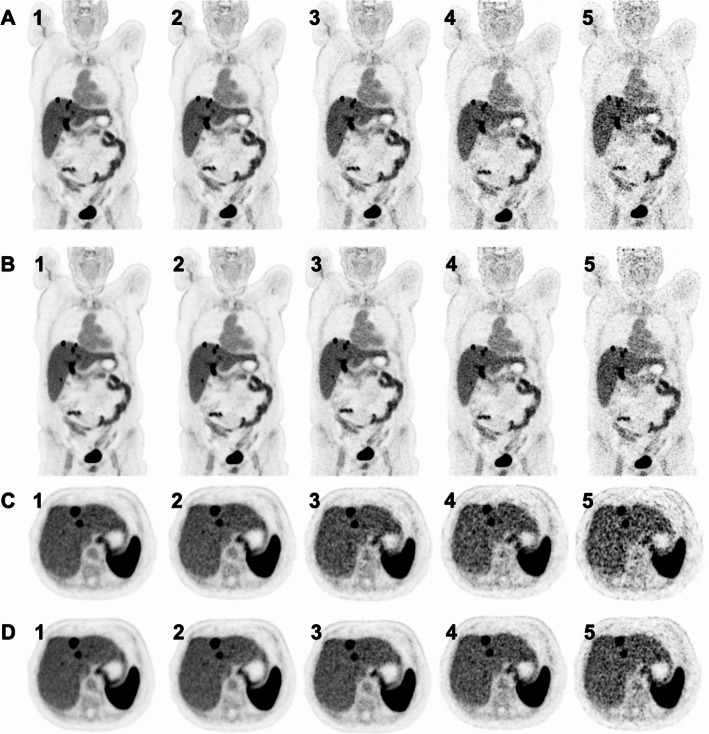
Axial and coronal [^18^F]SiTATE PET of a 5 min scan in HS mode (**A/C**) and UHS mode (**B/D**) of a 78 years old female patient with a metastasized ileum NET G2. **1**: 3.0 MBq/kg; **2**: 2.0 MBq/kg; **3**: 1.0 MBq/kg; **4**: 0.5 MBq/kg; **5**: 0.25 MBq/kg

To simulate reduced activities of injected [^18^F]SiTATE (2.0 MBq/kg, 1.0 MBq/kg, 0.5 MBq/kg, and 0.25 MBq/kg), PET listmode data of the 5 min emission scan at 3.0 MBq/kg were rebinned for shorter frame durations. Image reconstruction was performed using an investigational software prototype (e7 tools, Siemens Healthineers). This software allowed the use of either all events stored in the listmode data or just a subset of events. The subset included only events acquired within a limited axial acceptance angle of 18°, referred to as high sensitivity (HS) mode. In contrast, the full event dataset, acquired with the full 52° acceptance angle, was referred to as ultra-high sensitivity (UHS) mode. For more details on the sensitivity modes and acceptance angles of the Biograph Vision Quadra, refer to Schmidt et al. [19]. An example of a reconstructed [^18^F]SiTATE PET simulating lower activities in both HS and UHS mode is provided in Fig. 1.

### PET image analysis

Image analysis was performed by two readers in consensus reading for all scans, starting first with the standard activity and then the reduced activities in random order. The dedicated software SyngoVia^®^ (Version 2.3.1, Siemens Healthineers; Knoxville, TN, USA) and the Affinity Hybrid Viewer (Version 3.0.5, Hermes Medical Solution, Sweden) were used. To reduce over-representation of a specific patient or organ system, a maximum of 10 representative lesions per patient and a maximum of 5 lesions per organ site were analyzed in patients with multifocal or disseminated disease. To enhance the accuracy of the selection process, the largest and smallest lesions within each organ system were chosen, while the remaining lesions were selected randomly. The lesion characterization was performed by a nuclear medicine and a radiologist in combination with arterial and venous phase CT imaging to avoid false-positive lesions being classified as malignant.

Subjective overall image quality and image noise as well as conspicuity of SSTR positive lesions were assessed using a 5-point Likert scale, as previously described [21]:

- *image quality*: state-of-the-art quality (Likert score 5), superior to average (4), regular quality of daily practice (3), barely diagnostic (2), or non- diagnostic (1).
- *image noise*: near-imperceptible noise (5), lower than regular image of daily practice (4), similar to regular image of daily practice (3), increased noise, slightly worse than regular image of daily practice (2), excessive noise (1).
- *lesion detectability*: well-defined (5), fairly defined (4), hazy, but recognizable (3), ill-defined, impairing diagnostic confidence (2), un-recognizable (1).

Lesion uptake was quantified by measuring the SUV_mean_ and SUV_SD_ in a volume of interest (VOI) using a semi-automated rendering at 50% maximum threshold. A 15 cm^3^ spherical VOI was used to measure background uptake in the right hepatic lobe’s healthy tissue, as previously described [22]. VOIs were manually segmented in the standard scan (3.0 MBq/kg, 5 min, HS mode) and consecutively overlayed into the simulated scans. The tumor-to-liver ratio (TLR) was calculated as previously described [23]. The MTV was calculated by a threshold-based semi-automatic volumetric segmentation using the software tool Affinity Hybrid Viewer (Version 3.0.5, Hermes Medical Solution, Sweden). The threshold of 1.5 × SUV_mean_ + 2 × SD (standard deviation) of healthy liver tissue was chosen as previously described [4, 6, 24]. Only patients with MTV greater than 5 ml were included in the analysis in order to eliminate artifactual findings associated with very small volumes. The coefficient of variation (CoV) was calculated to assess objective image noise, as previously published [22]. A CoV of 15% is recommend by the European Association of Nuclear Medicine (EANM) and the European Federation of Organisations for Medical Physics (EFOMP) [25]. The signal-to-noise ratio (SNR) was defined as the reciprocal of the CoV [21].

### Statistical analysis

Statistical analysis was performed using GraphPad prism (Version 9.4.1, GraphPad Software, San Diego, CA, USA). A two-way ANOVA with Bonferroni correction was performed to assess differences in the SUV, the MTV and the SNR of the simulated reduced administered activities and sensitivity modes in comparison to the standard clinical scan (3 MBq/kg, 5 min).

### IEC phantom experiment and evaluation

Image quality, noise and contrast recovery were assessed via experiments with a standard National Electrical Manufacturers Association (NEMA) IEC phantom [26]. The phantom was filled with [^18^F]-FDG with an activity concentration of 3.3 kBq/ml in the background compartment and a sphere-to-background ratio of 4:1.

The phantom was positioned such that the lung insert was aligned with the transaxial center of the FOV and the spheres were aligned with the axial center of the Biograph Vision Quadra scanner.

In accordance to the patient study, different frame durations were employed to simulate reduced injected doses, equivalent to a 5 min scan with activity concentrations corresponding to 3, 2, 1, 0.5, and 0.25 MBq/kg with [^18^F]SiTATE at 90 min p.i.

Image reconstruction utilized the UHS mode and was the same as described for the patient study, with attenuation correction performed with a diagnostic CT scan (120 kVp tube potential, automatic tube current modulation with 210 mAs ref.). In addition to the 4 iterations and 5 subsets (4i5s), which is the standard setting for scans with [^18^F]SiTATE at our institution, reconstructions were performed with 2i5s, 3i5s and 5i5s. The evaluation of the impact of varying injected doses and number of iterations was performed as follows. Visually image quality (IQ) of transversal views was assessed in combination with the CoV as a metric for image noise as well as with contrast recovery coefficients (CRCs) as metric to resolve different sized tumors.

The CoV was determined as the ratio of the standard deviation to the mean value of the voxel values of a box shaped VOI in the background (in-plane dimensions of 150 × 15 mm^2^ and an axial dimension of 170 mm).

CRCs were calculated using the formula according to NEMA NU 2-2018 [26], with the activity concentrations of the spheres derived from the mean voxel values of spherical VOIs with diameters of 37-, 17- and 10-mm.

## Results

### Subjective image quality and noise

**Fig. 2:**
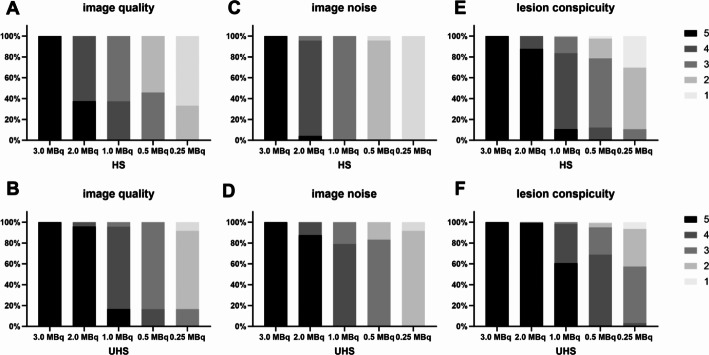
Subjective image quality ratings according to Likert-Score. Overall image quality (**A**: HS; **B**: UHS), image noise (**C**: HS; **D**: UHS) and lesion conspicuity (**E**: HS; **F**: UHS)

PET image quality of the standard clinical scan at 3.0 MBq/kg was rated as state-of-the-art quality with a mean Likert score of 5.0 ± 0 in both the HS and UHS modes. Reduction of administered activity led to a decrease in image quality beginning at 2 MBq/kg. Images in UHS mode were generally rated superior compared to images in HS mode at the same administered activity (Fig. 2A/B).

Image noise levels generally increased with decreasing activity. At a administered activity of 0.5 MBq/kg in HS mode, noise was rated as worse than that of reconstructed PET images in routine practice. As demonstrated for overall image quality, the Likert scores for image noise were superior in the UHS mode compared to the HS mode (Fig. 2C/D). Additionally, in order to quantitatively characterize image noise, we calculated the CoV and SNR.

### Lesion detectability

In total, *n* = 122 lesions were detected in the standard clinical scan (3 MBq/kg, 5 min, HS mode) and included in this analysis (64 liver metastases, 27 lymph node metastases, 13 bone metastases, 14 peritoneal lesions and 4 primary tumors in the pancreas). With a reduction of the administered activity of approximately 66% to 1.0 MBq/kg, all lesions remained detectable in both HS and UHS modes. With further reduction of the administered activity to 0.5 MBq/kg and 0.25 MBq/kg, the lesion detection rate decreased to 98% and 70% (HS) and 99% and 93% (UHS), respectively (Table 2).

**Table 2:** Lesion detection rate according to injected activity and sensitivity mode

[^18^F]SiTATE MBq/kg	Sensitivity mode	Number of lesions	Detection rate
3.0	HS	122	100%
3.0	UHS	122	100%
2.0	HS	122	100%
2.0	UHS	122	100%
1.0	HS	122	100%
1.0	UHS	122	100%
0.5	HS	119	98%
0.5	UHS	121	99%
0.25	HS	85	70%
0.25	UHS	114	93%

### Objective image quality

**Fig. 3:**
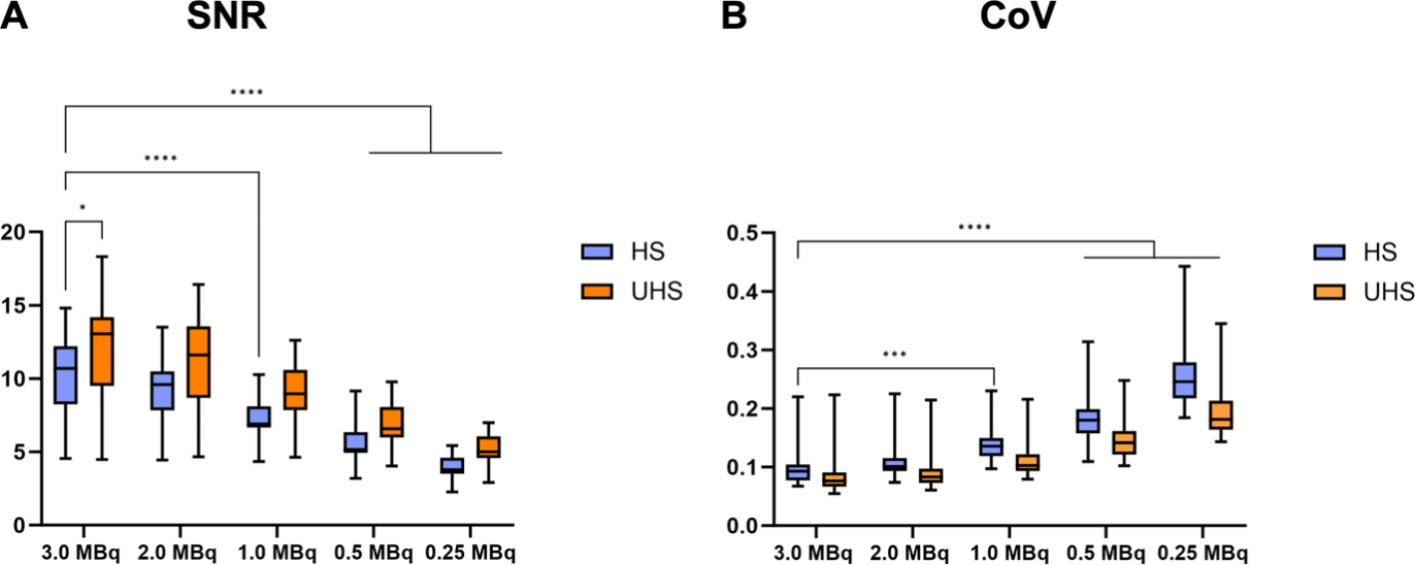
SNR (**A**) and CoV (**B**) of the liver background according to simulated dose and sensitivity mode. All data are presented as boxplot showing the median value (central line) and the 25–75th percentiles. Whiskers represent the maximum and minimum and are considered as significant at *p* < 0.05 (*), *p* < 0.01 (**), *p* < 0.001 (***) and *p* < 0.0001 (****)

Details of CoV and SNR measurements are provided in Table 3; Fig. 3. The recommended CoV of less than 15% was achieved for all scans with an administered activity of 1.0 MBq/kg or more (both HS and UHS). Furthermore, a CoV of 15% was achieved at 0.5 MBq/kg in UHS mode (Fig. 3B). The CoV was consistently lower in UHS mode compared to the HS mode. Generally, CoV values in the HS mode were comparable to the CoV values in the UHS mode but at half of the administered activity. For instance, a CoV of 14.1% was observed at 1.0 MBq/kg in HS mode, while a CoV of 15.0% was measured at 0.5 MBq/kg in UHS mode.

**Table 3:** CoV and SNR mean values and standard deviation according to simulated dose and sensitivity mode

[^18^F]SiTATE MBq/kg	Sensitivity mode	CoV mean ± SD in %	Mean SNR ± SD
3.0	HS	10.2 ± 3.8	10.4 ± 2.7
3.0	UHS	8.8 ± 3.9	12.4 ± 3.5
2.0	HS	11.1 ± 3.4	9.4 ± 2.2
2.0	UHS	9.5 ± 3.7	11.2 ± 3.0
1.0	HS	14.1 ± 3.5	7.3 ± 1.5
1.0	UHS	11.45 ± 3.4	9.0 ± 2.1
0.5	HS	18.6 ± 4.6	5.6 ± 1.3
0.5	UHS	15.0 ± 3.9	6.8 ± 1.5
0.25	HS	25.5 ± 5.9	4.0 ± 0.8
0.25	UHS	19.5 ± 4.7	5.2 ± 1.1

The SNR gradually decreased with reduction of the administered activity (Table 3). At 1.0 MBq/kg, the mean SNR was 7.3 ± 1.5 (HS) and 9.0 ± 2.1 (UHS). As shown for the CoV, an improvement in SNR was observed for reconstructions with the UHS mode compared to the HS mode. Regarding the SNR, a two-way ANOVA main effect analysis revealed that the simulated activity reduction (*p* < 0.01) and the sensitivity mode (*p* < 0.01) independently affected the SNR significantly. The post-hoc multiple comparison test revealed statistically significant differences in the mean SNR between the 3.0 MBq/kg standard scan in HS mode and in UHS mode for both sensitivity modes at 0.5 and 0.25 MBq/kg as well as for 1.0 MBq/kg in HS mode (Fig. 3A).

### Quantitative PET parameters

**Fig. 4:**
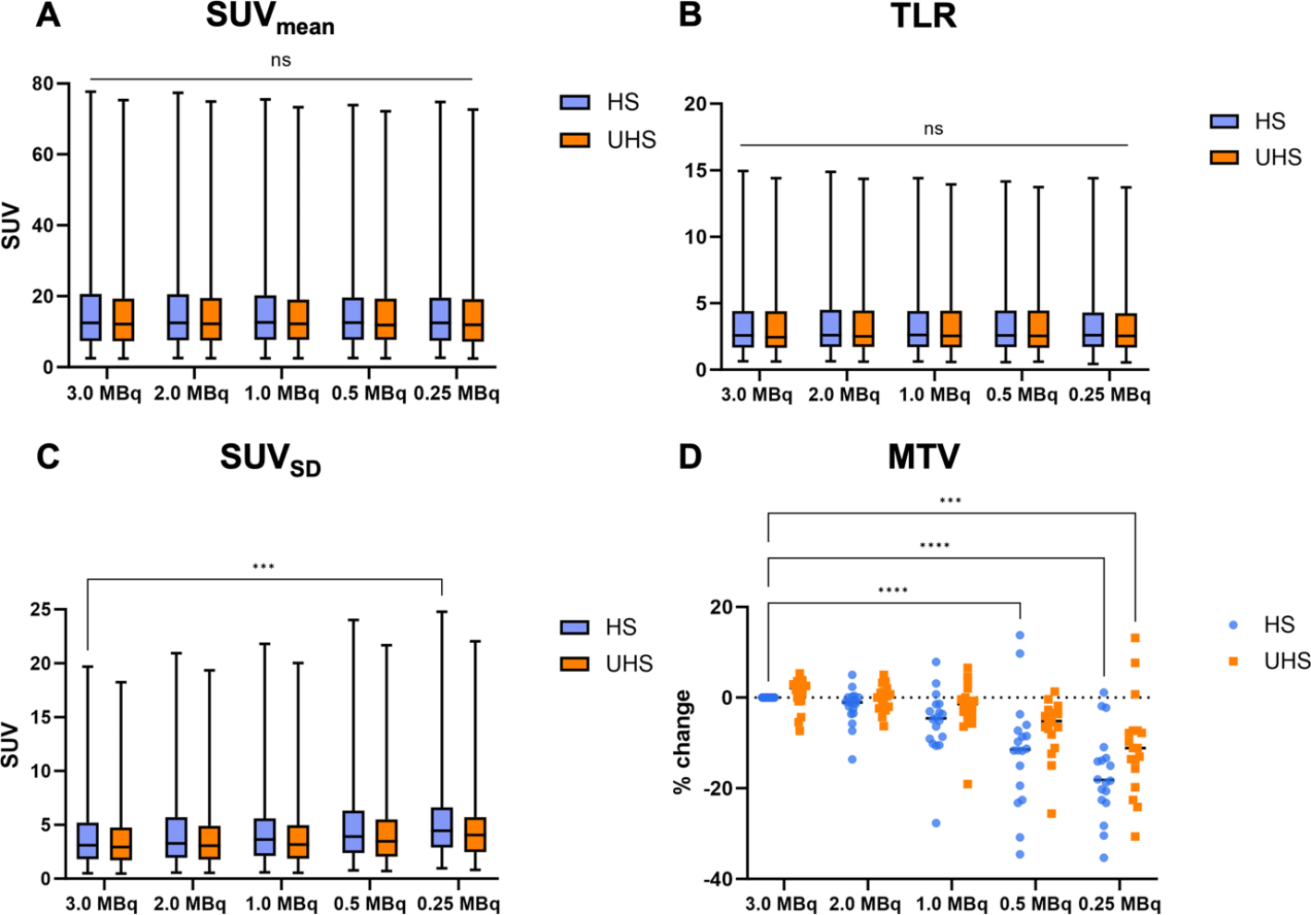
SUV_mean_ (**A**), TLR (**B**) and SUV_SD_ (**C**) values for all lesions. Percentage changes in the MTV in comparison to the standard scan with 3 MBq/kg in HS mode. All data are presented as boxplot showing the median value (central line) and the 25–75th percentiles. Whiskers represent the maximum and minimum and are considered as significant at *p* < 0.05 (*), *p* < 0.01 (**), *p* < 0.001 (***), *p* < 0.0001 (****) and as not significant (ns)

Details of quantitative PET measurements are shown in Fig. 4. Generally, only minimal changes in SUV_mean_ and TLR were detected with reduced activities in both HS and UHS mode. Results of the two-way ANOVA indicate that there was no significant interaction between the effects of the sensitivity mode and the simulated administered activity reduction for the SUV_mean_ (*p* > 0.99) and the TLR (*p* > 0.99) (Fig. 4A/B). However, reduced administered activities led to an increase in SUV_SD_, which was found to be less pronounced in the UHS mode compared to the HS mode. For example, at 0.5 MBq/kg, an increase of 22.5% was observed in SUV_SD_ in the HS mode but only 9.7% in the UHS mode (Fig. 4C).

**Fig. 5:**
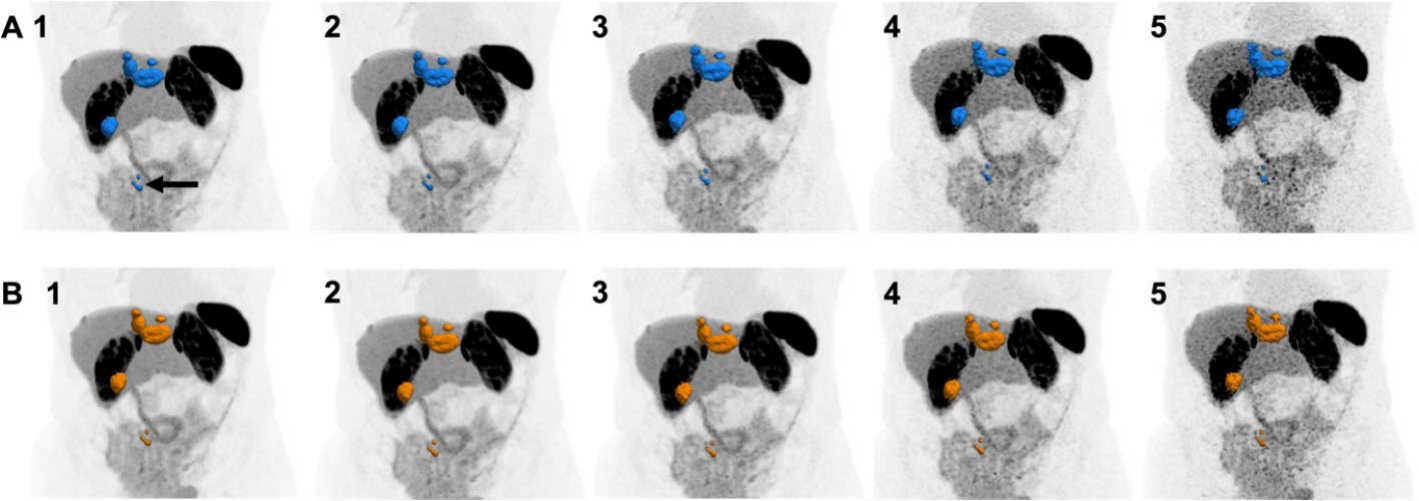
[^18^F]SiTATE PET reconstructions of a 5 min scan in HS mode (**A in blue**) and UHS mode (**B in orange**) of a 67 years old female patient with a hepatic and lymphogenic metastasized small intestine NET G2. With reduced injected activity, the decrease in the MTV can also be recognized, particularly in the area of the mesenteric lymph nodes (arrow). **1**: 3.0 MBq/kg; **2**: 2.0 MBq/kg; **3**: 1.0 MBq/kg; **4**: 0.5 MBq/kg; **5**: 0.25 MBq/kg

Figure 5 shows an example of the differences in MTV evaluation with administered activity reduction in both modes. Generally, a decrease in the activity of radiotracer administered resulted in a decrease in MTV (see also Fig. 4), which was more pronounced for the HS mode compared to the UHS mode. The two-way ANOVA main effect analysis showed that activity reduction simulation (*p* < 0.01) and the sensitivity mode (*p* < 0.01) independently had a statistically significant effect on the MTV. The post-hoc multiple comparison test revealed significant differences in the mean values of MTV between the 3.0 MBq/kg standard scan in HS mode and 0.5 MBq/kg in HS mode (*p* < 0.01) and for 0.25 MBq/kg in both sensitivity modes (UHS: *p* = 0.01; HS: *p* < 0.01) (Fig. 4D).

### IEC phantom image quality, noise and contrast recovery

**Fig. 6:**
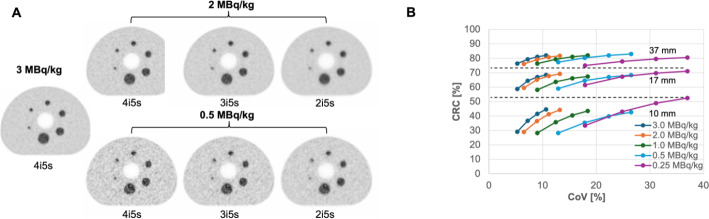
**A**: Transaxial view of the central slice of the IEC Phantom with ^18^F activity concentrations equivalent to an injected activity of 0.5, 2.0 and 3.0 MBq/kg and a 5 min scan. Further, comparison of the standard setting with 4 iterations and 5 subsets (4i5s) in comparison to 3i5s and 2i5s; **B**: Contrast Recovery (CRC) determined for the 10-, 17- and 37-mm spheres of the IEC phantom as a function of the coefficient of variation (CoV). Values are reported for activity concentrations equivalent to the patient examinations (3.0, 2.0, 1.0, 0.5 and 0.25 MBq/kg) and iterations from 2i5s-5i5s. Each point represents one iteration setting with the number of iterations increasing from left to right

As shown in Fig. 6A, while with an activity concentration of 2 MBq/kg still comparable image quality to 3 MBq/kg is obtained, a significant decline in image quality is observed for 0.5 MBq/kg. In particular at this lower activity concentration, a subjective improvement in image noise can be obtained by reducing the number of iterations.

The CRC degraded due to the partial volume effect towards smaller sphere sizes. For example, at 3 MBq/kg and 4i5s, CRC values were 81.1%, 67.1% and 41.6% for the 37-, 17- and 10-mm sphere, respectively.

The recovery could be maintained towards lower simulated activity concentrations with comparable CRC values, e.g., for 4i5s the CRC values were 81.1%| 82.0% (3 MBq/kg| 0.5 MBq/kg), 67.1%| 67.0% and 41.6%| 39.9% for the 37-, 17- and 10-mm sphere respectively.

Decreasing the number of iterations helped to mitigate image noise, e.g., at 0.5 MBq/kg the CoV values were 12.9%, 17.8%, 22.3% and 26.5% for 2i5s, 3i5s, 4i5s and 5i5s, respectively. However, this reduction in noise was accompanied by a decline in contrast recovery. At 0.5 MBq/kg, CRC values decreased with fewer iterations, such as 82.0%| 77.3% (4i5s| 2i5s), 67.0%| 59.0% and 39.9%| 28.3% for the 37-, 17- and 10-mm sphere, respectively.

All calculated CRCs and CoV values for the different simulated injected activities and over the different number of iterations can be found in Supplementary Table 3.

## Discussion

SSTR PET imaging is increasingly used and nowadays plays a crucial role in the clinical management of patients with NETs. [^68^Ga]Ga-DOTA-TOC was in 2019 the first SSTR PET approved by the Food and Drug Administration (FDA) [27]. SSTR PET imaging in patients with NETs using ^68^Ga labelled radiotracers is also recommended in the current guidelines of the European Neuroendocrine Tumor Society (ENETS) and the European Society of Medical Oncology (ESMO) [3, 28, 29]. To date, ^18^F-labeled SSTR radiotracers are not yet widely available, but it can be assumed that the increasing demand for SSTR imaging, not only for NETs but also for meningiomas, will require greater availability of SSTR tracers, which cannot be achieved with ^68^Ga labeled tracers. However, ^18^F-labelled radiotracers are currently not cited in the SSTR PET imaging guidelines of the EANM and SNMMI [30]. Furthermore, these tracers have been shown to be promising in NETs and meningiomas [20, 23, 31], due to the lower mean positron range of ^18^F and the resulting better spatial resolution compared with ^68^Ga. We assume that most specialized PET centers currently offering 18F-labelled SSTR imaging inject a standard administered activity of 3 MBq [18F]SiTATE per kilogram body weight and scan patients in a SAFOV PET scanner [13, 20].

Recently introduced LAFOV PET/CT scanners are characterized by their substantially increased sensitivity [17, 18]. A significant activity reduction of the injected radiotracer and/or a reduction of PET scan time is possible in LAFOV PET scanners, while preserving clinically diagnostic image quality [14, 15]. One study conducted in patients with malignant melanoma utilizing the Siemens Biograph Vision Quadra showed that an activity reduction to 2.0 MBq/kg [^18^F]FDG with a 5-minute PET scan did not result in deterioration of clinical image quality [14]. Another study utilizing the uEXPLORER system (United-imaging, Houston, USA) showed that the acquisition time for [^68^Ga]Ga-FAPI-04 scans could be reduced down to 2 min from 5 min without deterioration of the image quality, even in the case of the injection of only half the tracer dosage [15].

Our results indicate that an activity reduction of 66% down to 1 MBq/kg is feasible in a LAFOV PET/CT scanner, thereby maintaining high diagnostic image quality without statistically significant deterioration of image quality or semi-quantitative PET uptake parameters including MTV.

Regarding subjective PET image quality at reduced administered activities of [^18^F]SiTATE, the recently introduced UHS mode showed improved quality in all categories compared to the standard HS mode.

A CoV of < 15%, as recommended by EANM and EFOMP, was achieved with a reduction of the administered activity down to 1.0 MBq/kg in both the HS and UHS modes [25]. There were no significant differences in the SUV_mean_ and TLR values for both the different administered activities and sensitivity modes. However, the semi-quantitative parameters showed an increase in the standard deviation (e.g. SUV_SD_), which coincided with a decrease in the SNR. Higher SUV_SD_ of the lesions and in the background VOI of the liver had a direct impact on the quantification of the MTV, since the calculation of MTV is based on the SUV_mean_ and SUV_SD_ of the liver. No significant changes in MTV were seen with reduced administered activities down to 1.0 MBq/kg. However, with further reduction of the administered activity down to 0.5 MBq/kg and 0.25 MBq/kg, a significant reduction of the MTV in comparison to the standard clinical scan at 3 MBq/kg was observed. Very low activity [^18^F]SiTATE PET examinations with these dosages are therefore not recommended in clinical practice, since they might alter the assessment of treatment response, for example, in the context of peptide receptor radionuclide therapy (PRRT).

To evaluate whether towards lower activities an improvement in image quality and quantification - and thus potentially better lesion conspicuity - can be achieved by adjusting the number of iterations in the image reconstruction, we performed the IEC phantom study. The advantage of the phantom model is the possibility of a standardized assessment with known ground truth on contrast recovery and different tumor sizes mimicked by spherical inserts.

Our findings demonstrate that reducing the number of iterations allows for improved image quality in terms of reduced image noise at lower activity levels; such as for 0.5 MBq/kg a CoV of 12.9% (2i5s) versus 22.3% (4i5s)– in comparison the full activity reference was 9.0% at 3 MBq/kg (4i5s). However, this noise reduction is accompanied by a decline in contrast recovery, which serves as an indicator for diminished lesion detectability and quantification accuracy. This effect was particularly pronounced for smaller structures, with the 10 mm sphere showing a decrease in CRC from 39.9% (4i5s) to 28.3% (2i5s) at 0.5 MBq/kg. In cases where resolving small lesions is less critical, such as for post radioembolization therapy dosimetry [32], a lower iteration setting may be preferred to enhance subjective image quality and reduce noise. In a clinical context, this issue may be more challenging than image quality itself, particularly in NETs. The detection of small, early-stage organ metastases would require a change in the overall management strategy compared to a scenario with only locoregional involvement. In conclusion, here maintaining the standard iteration setting of 4i5s is favorable even for lower activity levels to avoid compromising contrast recovery and thus potentially lesion detectability and quantification.

As mentioned above, our study showed that the recently introduced UHS mode provided improved clinical performance in low administered activity examinations. Due to its substantially (i.e., two-fold) higher sensitivity, the UHS mode might enable a more widespread clinical application of ^18^F-labelled SSTR tracers, even in the case of lower tracer yield of [^18^F]SiTATE or if the tracer has to be delivered to distant PET centers.

### Limitations

The major limitation of this study was the small study cohort of 24 patients. Furthermore, no children or young adults have been included in this study. However, in our patient cohort we analyzed a total of 122 lesions. Furthermore, 109 of 122 lesions were located in the abdominal region, which is in the center of the FOV in LAFOV scanners and previous studies have demonstrated that the UHS mode provides superior performance in the center of the FOV [19]. Therefore, the addition of the UHS mode could be particularly beneficial for this group of patients. A key limitation was the order in which the images were analyzed, as the lesions were selected using the standard activity PET/CT. This selection process could be a potential bias for the readers in the analysis of the reduced activities.

In this study, the reduction of the injected activity was simulated in order to achieve better intra-individual comparability. However, clinical scans (Supplementary Table 2), in which reduced activities had to be used due to low production yields, were able to confirm the semi-quantitative values (CoV, SNR) determined by our simulation. Also, it should be noted that this study should not be generalized to other SSTR-positive tumors which might be associated with a different metastatic pattern. A further limitation of this work focusing on activity reduction is that it cannot be generalized to other SSTR-tracers with higher positron energy such as ^68^Ga or tracers with longer half-lives such as ^64^Cu.

## Conclusion

In conclusion, this study demonstrated that the injected activity of [^18^F]SiTATE can be reduced by 66% down to 1 MBq/kg in a LAFOV PET/CT scanner by maintaining the acquisition time at 5 min, with no meaningful impact on image quality, lesion detectability, or quantitative PET parameters. Furthermore in low activity [^18^F]SiTATE PET/CT, the recently introduced UHS mode improves image quality and noise as well as lesion detectability compared to HS mode, further reinforcing the clinical benefits of this reconstruction mode. Overall, this may contribute to more consistent PET/CT examination planning and execution in spite of variable tracer production.

## Electronic supplementary material

Below is the link to the electronic supplementary material.

Supplementary Material 1

## Data Availability

The datasets used and/or analyzed during the current study are available from the corresponding author on reasonable request.
