# Supplementary material for: Low-activity [18F]-somatostatin receptor (SSTR) imaging using [18F]SiTATE on a long axial field-of-view PET/CT scanner
Source: EJNMMI Phys. 2025 Feb 5;12:13. doi: 10.1186/s40658-025-00720-z (PMC11799470; doi:10.1186/s40658-025-00720-z)
Supplement: Supplementary file 1 — Supplementary Material 1 [file 40658_2025_720_MOESM1_ESM.docx]

**Supplementary Table 1.** Additional patient information

| Patient ID | Age | Sex | Weight (kg) | Height (cm) | BMI | Activity |
| --- | --- | --- | --- | --- | --- | --- |
| 1 | 53 | W | 59.6 | 160 | 23.3 | 203 |
| 2 | 69 | W | 49.3 | 165 | 18.1 | 161 |
| 3 | 57 | W | 58.6 | 172 | 19.8 | 198 |
| 4 | 59 | M | 93.9 | 182 | 28.3 | 338 |
| 5 | 51 | W | 61.8 | 165 | 22.7 | 184 |
| 6 | 62 | M | 92 | 177 | 29.4 | 290 |
| 7 | 57 | M | 51.2 | 165 | 18.8 | 185 |
| 8 | 73 | M | 84.3 | 172 | 28.5 | 291 |
| 9 | 72 | M | 88 | 184 | 26 | 287 |
| 10 | 78 | W | 67.2 | 165 | 24.7 | 218 |
| 11 | 43 | W | 54 | 163 | 20.3 | 187 |
| 12 | 79 | M | 67.3 | 173 | 22.5 | 210 |
| 13 | 59 | M | 73.5 | 171 | 25.1 | 263 |
| 14 | 45 | M | 93.5 | 174 | 30.9 | 302 |
| 15 | 82 | M | 73.6 | 177 | 23.5 | 244 |
| 16 | 63 | M | 85 | 170 | 29.4 | 260 |
| 17 | 62 | W | 54.6 | 157 | 22.2 | 206 |
| 18 | 88 | W | 112.7 | 168 | 39.9 | 349 |
| 19 | 79 | W | 59.5 | 157 | 24.1 | 212 |
| 20 | 77 | W | 62.9 | 160 | 24.6 | 203 |
| 21 | 67 | W | 62 | 160 | 24.2 | 190 |
| 22 | 83 | W | 74.6 | 158 | 29.9 | 252 |
| 23 | 79 | M | 74.3 | 171 | 25.4 | 228 |
| 24 | 64 | W | 68 | 168 | 24.1 | 249 |

**Supplementary Table 2.** Data of the four patients with lower injected activity.

| Patient ID | Primary tumor site | Age | Activity  MBq/kg BW | CoV in % HS | CoV in % UHS | SNR  HS | SNR  UHS |
| --- | --- | --- | --- | --- | --- | --- | --- |
| 25 | Pancreas | 82 | 0.8 | 14.3 | 13.7 | 7.0 | 7.3 |
| 26 | Pancreas | 78 | 0.9 | 15.1 | 14.3 | 6.6 | 7.0 |
| 27 | Ileum | 81 | 0.8 | 16.9 | 14.8 | 5.9 | 6.7 |
| 28 | Pancreas | 63 | 0.9 | 15.1 | 14.5 | 6.6 | 6.9 |

**Supplementary Table 3:** Coefficient of variation (CoV) and Contrast Recovery (CRC) for the 10-. 17- and 37-mm spheres of the IEC phantom for activity concentrations equivalent to patient examinations (3.0, 2.0, 1.0, 0.5 and 0.25 MBq/kg) and iterations from 2i5s-5i5s.

|  | CoV [%] | | | | CRC (10 mm) [%] | | | | CRC (17 mm) [%] | | | | CRC (37 mm) [%] | | | |
| --- | --- | --- | --- | --- | --- | --- | --- | --- | --- | --- | --- | --- | --- | --- | --- | --- |
| simulated injected activity [MBq/kg] | 2i5s | 3i5s | 4i5s | 5i5s | 2i5s | 3i5s | 4i5s | 5i5s | 2i5s | 3i5s | 4i5s | 5i5s | 2i5s | 3i5s | 4i5s | 5i5s |
| 3.00 | 5.3 | 7.2 | 9.0 | 10.6 | 29.1 | 36.7 | 41.6 | 44.6 | 58.8 | 64.4 | 67.1 | 68.5 | 76.4 | 79.4 | 81.1 | 82.1 |
| 2.00 | 6.5 | 8.9 | 11.2 | 13.2 | 29.0 | 36.5 | 41.2 | 44.2 | 59.6 | 65.2 | 67.8 | 69.2 | 76.2 | 79.1 | 80.8 | 81.8 |
| 1.00 | 9.0 | 12.4 | 15.5 | 18.4 | 28.3 | 35.7 | 40.5 | 43.5 | 58.2 | 63.7 | 66.1 | 67.4 | 76.4 | 79.3 | 81.0 | 82.0 |
| 0.50 | 12.9 | 17.8 | 22.3 | 26.5 | 28.3 | 35.4 | 39.9 | 42.6 | 59.0 | 64.6 | 67.0 | 68.3 | 77.3 | 80.3 | 82.0 | 83.0 |
| 0.25 | 17.9 | 24.8 | 31.1 | 37.0 | 33.4 | 43.0 | 48.9 | 52.5 | 61.4 | 67.2 | 69.8 | 71.1 | 75.0 | 77.9 | 79.5 | 80.5 |
